# MicroRNAs in blood act as biomarkers of colorectal cancer and indicate potential therapeutic targets

**DOI:** 10.1002/1878-0261.13065

**Published:** 2021-08-05

**Authors:** Axel Stang, Hauke Weilert, Michael J. Lipp, Karl J. Oldhafer, Jörg D. Hoheisel, Chaoyang Zhang, Andrea S. Bauer

**Affiliations:** ^1^ Department of Haematology, Oncology & Palliative Care Asklepios Hospital Barmbek Hamburg Germany; ^2^ Faculty of Medicine Semmelweis University Hamburg Germany; ^3^ Department of Abdominal & Visceral Surgery Asklepios Hospital Barmbek Hamburg Germany; ^4^ Division of Functional Genome Analysis German Cancer Research Center Heidelberg Germany

**Keywords:** bioinformatics, colorectal cancer, metastases, microarray, microRNA, pathway analysis

## Abstract

Association studies have linked alterations of blood‐derived microRNAs (miRNAs) with colorectal cancer (CRC). Here, we performed a microarray‐based comparison of the profiles of 2549 miRNAs in 80 blood samples from healthy donors and patients with colorectal adenomas, colorectal diverticulitis and CRC at different stages. Confirmation by quantitative real‐time PCR (RT‐PCR) was complemented by validation of identified molecules in another 36 blood samples. No variations in miRNA levels were observed in samples from patients with colorectal adenomas and diverticulitis or from healthy donors. However, there were 179 CRC‐associated miRNAs of differential abundance compared to healthy controls. Only three – miR‐1225‐5p, miR‐1207‐5p and miR‐4459 – exhibited increased levels at all CRC stages. Most deregulated miRNAs (128/179, 71%) specifically predicted metastatic CRC. Pathway analysis found several cancer‐related pathways to which the miRNAs contribute in various ways. In conclusion, miRNA levels in blood vary throughout CRC progression and affect cellular functions relevant to haematogenous CRC progression and dissemination. The identified biomarker and therapeutic candidates require further confirmation of their clinical relevance.

## Graphical abstract

Here, we performed a comprehensive study on the abundance variations of 2549 miRNAs in blood from 80 healthy donors and patients with colorectal disease. We further validated the results in 36 independent samples by quantitative real‐time PCR. Patients with colorectal adenomas or diverticulitis and healthy donors exhibited no difference in their miRNA profiles, while patients with colorectal cancer displayed miRNAs with differential abundance in a stage‐specific manner. The miRNAs identified in our study contribute to several cancer‐related pathways relevant to colorectal cancer progression and haematogenous dissemination.

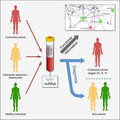

**Abbreviations**
CRA: Colorectal adenoma
CRC: Colorectal cancer
CRD: Colorectal diverticulitis
CTC: Circulating tumour cell
FC: Fold change
IPA: Ingenuity Pathway Analysis
mCRC: Metastatic colorectal cancer
miRNA: MicroRNAs
RMA: Robust Multichip Average
RT‐PCR: Quantitative real‐time PCR
UICC: Union Internationale Contre le Cancer

## 1 Introduction

Colorectal cancer (CRC) is a leading cause of cancer‐related death worldwide [1]. The 5‐year survival rate of nonmetastatic CRC (UICC stages I–III) ranges between 60% and 95% and falls to < 15% for patients with metastatic CRC (mCRC, UICC stage IV) [1, 2]. Thus, there is a need for developing improved CRC screening and treatment options to reduce CRC mortality [2, 3] MicroRNAs (miRNAs) are potent post‐transcriptional gene expression regulators and appear to be involved in virtually all features of CRC, including initiation, progression and its metastatic spread [4]. Association studies have linked alterations of blood‐derived miRNAs with CRC, but they assessed only a limited number of miRNAs with poor replicability of putative biomarker candidates across studies [5, 6]. Comprehensive assessments of miRNA abundance changes in blood from CRC patients are lacking. There are only studies reporting about analyses on some miRNA molecules each [5, 6, 7, 8, 9]. Also, the blood‐based miRNAs reported to be deregulated in CRC were not controlled for confounding effects by premalignant and/or inflammatory colorectal conditions, such as colorectal adenoma (CRA) or diverticulitis (CRD) [5, 9]. Moreover, miRNA profiles may diversify during CRC progression and reflect in blood the CRC progression level. No study has yet provided an assessment of miRNA alterations in blood at different CRC stages.

The human genome encodes over 2500 miRNAs, which function in complex regulatory networks, often in reciprocal interactions with the targets and pathways that they regulate [10]. Current technologies provide platforms to address this complexity as they enable detection of all known human miRNAs in a single assay [11]. While biomarker discovery focused on miRNAs with consistently altered abundance across CRC stages, distinct miRNA levels during progressive stages may reflect how CRC dissemination is represented in blood. Apart from the easier access to blood rather than tissue biopsies, blood samples may provide information regarding compartment‐related functions of miRNA alterations in haematogenous CRC dissemination. To gain an insight into the biological meaning of miRNA dysregulation in blood, bioinformatics tools that interrogate knowledge databases can help identifying affected targets and pathways at the level of large miRNA datasets [12]. Together, these methodologies may reveal previously unknown prometastatic effects of miRNA dysregulation in haematogenous spread and identify tumour vulnerabilities for the design of novel antimetastatic strategies.

In the present study, we used a human miRNA analysis microarray to compare blood profiles of 2549 miRNAs in healthy control donors and patients with CRD and CRA as well as CRC at different UICC stages (I/II, III and IV) with the aim of assessing candidate biomarker miRNAs for blood‐based diagnostic tests common to all CRC stages. We also tested for stage‐independent but CRC‐specific alterations. Further, we subjected to bioinformatics analysis the miRNAs that exhibited changed abundance levels in the blood for identifying potential target molecules and pathways that may be affected by particularly mCRC‐specific alterations in the miRNA profile.

## 2 Materials and methods

### 2.1 Ethics statement

Research was performed in accordance with the Declaration of Helsinki of the World Medical Association. The study was approved by the ethics committee of the Ärztekammer Hamburg (positive ethics vote PV4594 of 28 November 2013). All patients and healthy donors gave written informed consent to use their samples for research prior to their inclusion in the study.

### 2.2 Sample collection

The participants for this prospective exploratory single‐centre study were recruited at the Asklepios Hospital Barmbek in Hamburg, Germany, between January 2014 and December 2016 as well as between November 2020 and February 2021. The characteristics of the participants and the criteria for diagnosis and cohort inclusion are presented in Table 1. From each study participant, 5 mL peripheral blood was drawn into PAXgene Blood RNA tubes (BD, Franklin Lakes, USA). Blood samples were snap‐frozen in liquid nitrogen directly after sampling and subsequently stored at −80 °C until used for RNA isolation.

**Table 1:** Baseline characteristics of the study participants.

Participants	UICC stage	Cohort (no.)	Number (*n*)	Age (years) (mean ± SD) [range]	Gender male:female	Inclusion criteria	Laboratory values (at time of sampling)
Initial group of 80 study participants							
Healthy controls	–	1	16	58 ± 13.6 [39–84]	8 : 8	Colonoscopy (during the last 2 years): no CRC, CRA, or CRD clinic: no signs of malignancy or infection	Normal
Colorectal diverticulitis (CRD)	–	2	8	64 ± 10.1 [41–88]	4 : 4	Colonoscopy (≤ 3 months at follow up): CRD, no CRC or CRA Clinic: typical left‐sided abdominal pain; recovery under antibiotics; CT: typical signs of diverticulitis	Normal except parameters for inflammation
Colorectal adenoma (CRA)	–	3	8	57 ± 11.0 [28–89]	5 : 3	Colonoscopy (prior to polypectomy): CRA, no CRC or CRA polypectomy: CRA	Normal
Colorectal cancer (CRC)	I/II	4	16	61 ± 10.8 [35–85]	9 : 7	PT resection: CRC pT1‐4 pN0 R0; CT staging: cM0	Normal
Colorectal cancer (CRC)	III	5	16	64 ± 13.6 [32–88]	10 : 6	PT resection: CRC pT1‐4 ≥ pN1 R0; CT staging: cM0	Normal
Colorectal cancer (CRC)	IV	6	16	58 ± 12.8 [34–84]	9 : 7	Biopsy‐proven CRC CT staging: cT1‐4 cN0‐3 cM1a/b	Normal except liver values up to 2× normal
Validation group of 36 participants							
Healthy controls	–	7	10	52 ± 10.3 [38–77]	5 : 5	Colonoscopy (during the last 2 years): No CRC, CRA, or CRD Clinic: no signs for malignancy or infection	Normal
Colorectal cancer (CRC)	I/II	8	8	60 ± 9.6 [46–86]	4 : 4	PT resection: CRC pT1‐4 pN0 R0 CT staging: cM0	Normal
Colorectal cancer (CRC)	III	9	8	61 ± 10.2 [29–78]	5 : 3	PT resection: CRC pT1‐4 ≥ pN1 R0 CT staging: cM0	Normal
Colorectal cancer (CRC)	IV	10	10	56 ± 9.4 [34–73]	6 : 4	Biopsy‐proven CRC CT staging: cT1‐4 cN0‐3 cM1a/b	Normal except liver values up to ≤ 2‐fold of normal

CT, computed tomography; PT, primary tumour; SD, standard deviation; UICC, Union internationale contre le cancer.

### 2.3 RNA isolation and miRNA microarray analysis

RNA isolation was performed as described in detail earlier [13, 14, 15]. In short, 5 mL blood was centrifuged at 5000 g for 10 min at room temperature. Total RNA including miRNA from the cell pellet was isolated using the miRNeasy kit (Qiagen, Hilden, Germany) according to the manufacturer’s instruction. RNA integrity was evaluated using an Agilent 2100 Bioanalyzer (Agilent Technologies, Palo Alto, CA, USA). Only samples with an RNA integrity number of at least seven were used for further analyses. The total RNA prepared from individual samples was analysed at the Genomics Core Facility of the German Cancer Research Center (DKFZ) in Heidelberg on the Agilent Human miRNA Microarray Release 21.0 according to the detailed manufacturer’s protocol that is available from Agilent and includes process controls. Briefly, fluorescently labelled miRNA was prepared according to the Agilent miRNA Complete Labelling and Hyb Kit protocol. Hybridisation was performed in a SureHyb chamber (Agilent Technologies) at 55 °C for 20 h. Microarrays were scanned using the Agilent Scanner G2505C. After quantile normalisation, fold changes were calculated based on mean values of technical replicates. In total, 2549 human miRNAs were analysed for differences in their blood levels.

### 2.4 Statistical analysis

Statistical analyses were performed using the Chipster microarray data analysis software (http://chipster.csc.fi). After RMA normalisation and log_2_‐transformation of expression values, fold changes were calculated based on the mean values of the replicates. Single miRNA analyses for expression variations were carried out using t‐tests (unpaired, two‐tailed) for pairwise comparisons after verifying approximate normal distribution using empirical Bayes method. The resulting p‐values were adjusted for multiple testing using Benjamini–Hochberg’s adjustment. A log_2_‐fold change (FC) > 0.2 and an adjusted *P*‐value < 0.05 were considered as cut‐off criteria for significant up‐ or downregulation of differentially abundant miRNAs.

### 2.5 Evaluation by quantitative real‐time PCR

Each RNA sample was checked for genomic DNA contaminations that could obscure the measurement. One microgram of total RNA was converted to cDNA using the miScript Reverse Transcriptase mix (Qiagen) following the manufacturer’s instructions. Quantitative RT‐PCR was performed in triplicate with a LightCycler 480 instrument (Roche Diagnostics, Mannheim, Germany) using the miScript SYBR Green PCR Kit according to the manufacturer’s protocol. Molecules of the QuantiTect Primer Assays (Qiagen) were used as primers: hsa‐miR‐1225‐3p, hsa‐miR‐1260_1, hsa‐miR‐4516_1 and hsa‐RNU6‐2_11, the last used for normalisation purposes as suggested by Qiagen. Data were analysed using the lightcycler software (Roche Diagnostics).

### 2.6 Ingenuity pathway analysis

Bioinformatics analyses for all up‐ and downregulated miRNAs in the microarray dataset of mCRC patients in comparison with healthy controls were performed using the Ingenuity Pathway Analysis (IPA) software (Qiagen). Microarray‐based miRNA expression data were sorted by the log_2_‐fold change, uploaded into IPA for core analysis and then overlaid with the large‐scale molecular network implemented and available in the Ingenuity Knowledge Base. Downstream effect analyses were carried out to identify those cellular functions, targets, pathways and networks that are likely to be affected by the patterns of differentially abundant miRNAs. These analyses provided predictions about the activation state of each function, target, pathway and/or network, calculated as activation *Z*‐scores ([*Z*] > 2 is considered significant) [12].

## 3 Results

### 3.1 Study population

The initial study participants included healthy subjects as control group (cohort 1) and newly diagnosed patients with the premalignant and inflammatory colorectal conditions diverticulitis (CRD; cohort 2) or colorectal adenoma (CRA; cohort 3), as well as CRC at stages UICC I/II (cohort 4), UICC III (cohort 5) or UICC IV (cohort 6). They were recruited for this exploratory study between January 2014 and December 2016. The baseline characteristics of all participants and the criteria for diagnosis and cohort inclusion are presented in Table 1. Notably, the absence of CRC was confirmed by colonoscopy in all CRC‐free individuals (cohorts 1–3), and the presence of CRC was proven histologically in all CRC patients (cohorts 4–6). No treatment had been administered to any of the blood donors prior to sampling, specifically no antibiotic therapy in cohort 2 (CRD), no polypectomy in cohort 3 (CRA), no resection in cohorts 4 and 5 (CRC, UICC I/II and III) and no systemic therapy in cohort 6 (mCRC, UICC IV). From each study participant, peripheral blood was collected, snap‐frozen in liquid nitrogen directly after sampling and stored at −80 °C until used for RNA isolation. In the analysis, 80 samples met the strict quality requirements of the profiling analysis, including samples obtained from patients with CRA (*n* = 8), CRD (*n* = 8) and CRC at different stages (I/II, III and IV; for each group, *n* = 16) and from 16 healthy donors (Table 1).

### 3.2 miRNA level profiles

**Fig. 1:**
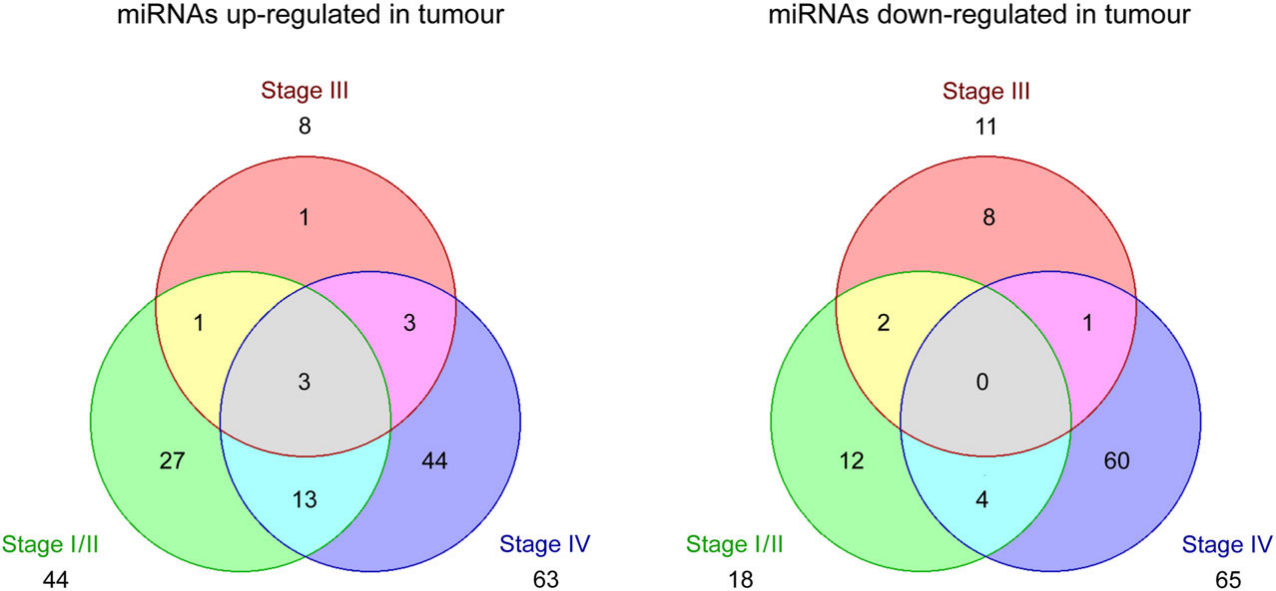
Venn diagram of the numbers of miRNAs with increased or decreased levels in the blood of colorectal cancer (CRC) patients of UICC stages I/II, III and IV, respectively, compared to miRNA levels in the blood of healthy donors. Only the three miRNAs – miR‐1207, miR‐1225 and miR‐4459 – exhibited higher levels in CRC patients at all stages.

**Fig. 2:**
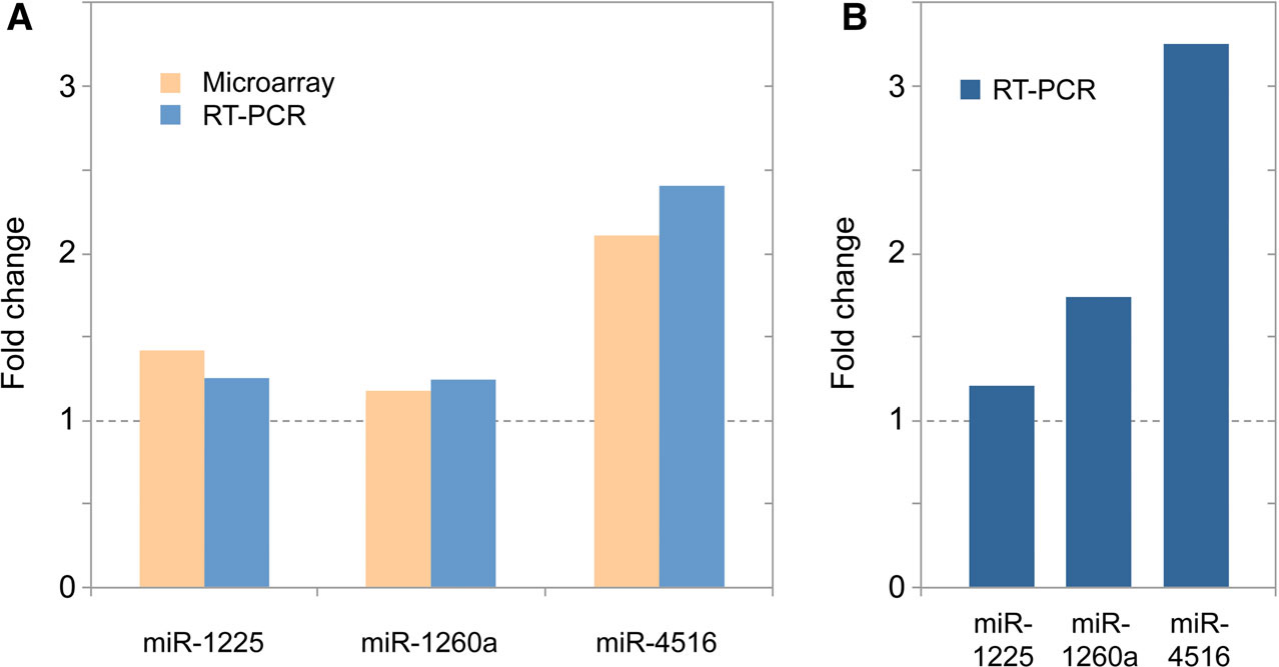
Confirmation and validation of expression variations by RT‐PCR. (A) For three miRNAs, RT‐PCR was performed with all 80 samples from tumour patients and healthy donors that were analysed on the microarray. The ratio of the mean transcript levels in all three tumour stages and healthy is shown for both the microarray analysis and the PCR assay. (B) For an independent validation of the results’ robustness, another set of 36 independent blood samples was collected and the isolated miRNA analysed by RT‐PCR.

Sample analysis followed a robust process that has been used in various analyses [13, 14, 15], including a study that looked at more than 1000 blood samples from patients with different cancers and noncancer diseases. Surprisingly, no significant variations in miRNA levels compared to the healthy controls (cohort 1) were detected in samples obtained from patients with colorectal adenoma and diverticulitis (cohorts 2, 3). This indicates that the identified CRC‐associated miRNA alterations were not confounded by premalignant or inflammatory colorectal conditions. By contrast, a total of 179 (7%) miRNAs with significantly changing levels (92 up and 87 down) were obtained from colorectal cancer patients (cohorts 4–6). There were actually rather large differences between UICC stages I/II, III and IV regarding the number and pattern of differentially abundant miRNAs in comparison with samples from healthy blood donors (Tables [Link], [Link], [Link], respectively). Most CRC‐associated deregulated miRNAs (71.5%, 128 of 179, 63 up and 65 down) were found at stage IV. The degree of specificity to UICC stages of the significantly varying miRNAs is documented by the small number of miRNAs that exhibited change at more than one stage (27 of 179; 15%; Fig. 1). Merely three miRNAs (1.7%, 3 of 179) – miR‐1225‐5p, miR‐1207‐5p and miR‐4459 – were detected to exhibit increased levels across all UICC stages. As a control, miR‐1225‐5p and two randomly selected deregulated miRNAs were analysed by RT‐PCR in all samples from healthy donors and tumour patients for confirmation of results obtained in the microarray analyses (Fig. 2A). To prove that the results hold true also in an independent set of samples, we performed RT‐PCR on miRNA isolated from blood of a second group of participants, who were recruited between November 2020 and February 2021. miRNA was isolated from blood of 8, 8 and 10 patients with stage I/II, stage III and stage IV CRC, respectively, as well as miRNA from 10 healthy blood donors (Table 1). Repeating the RT‐PCR, we obtained similar results, although a better discrimination could be observed (Fig. 2B).

### 3.3 Pathway analysis

**Fig. 3:**
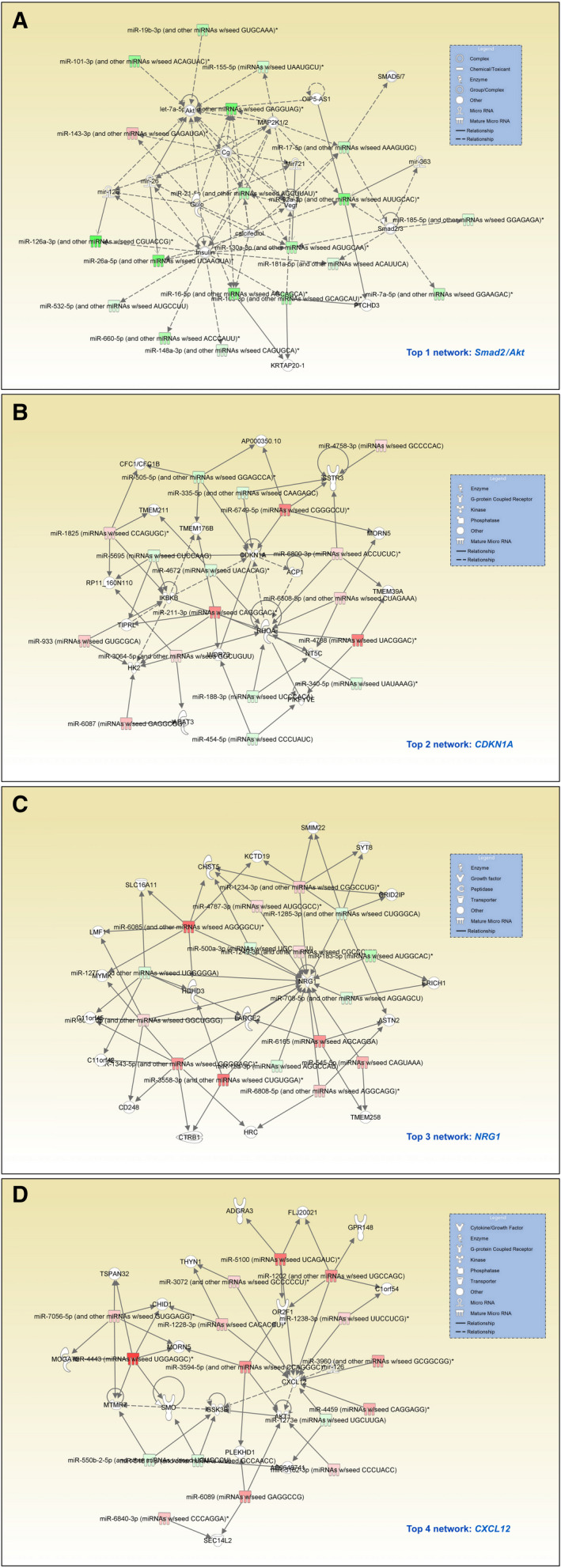
IPA interaction network maps of downstream effects of miRNAs deregulated in blood of metastatic colorectal cancer (mCRC) patients. The four top‐most networks are shown. Red or green colouring labels miRNAs of higher or lower abundance.

Ingenuity Pathway Analysis (IPA) was applied to the dataset of the stages I/II, III and IV in order to define pathways and molecules that might be affected by the observed changes in miRNA levels. Although less informative, we also performed a target scan for all miRNAs that exhibited variations in their abundance (Table S4). While stage I/II samples yielded several pathways that exhibit an association with cancer (Fig S1), only a single such network was identified analysing the stage III data (Fig S2). This result is not surprising, since far fewer miRNAs were found to be specific for stage III. Of particular interest was stage IV (mCRC). According to the number of specifically varying miRNAs, several pathways associated with cancer were identified, and they were also the most strongly affected ones (Table 2). Using existing functional annotations of the genes targeted by the miRNAs, the functions and diseases were defined, in which each network plays a role. A score was computed that indicates the relevance: a score of 2 is considered significant. Actually, the top scores obtained with the miRNA networks shown range from 22 up to 33 (Table 2), highlighting the importance to mCRC of the miRNA variations. Networks involving *SMAD2/AKT* (Fig. 3A) and *CDKN1A* (Fig. 3B) belong to ones that are affected by the observed miRNA patterns. The roles of *SMAD2/AKT* and *CDKN1A* in the progression of CRC are well established [16, 17, 18, 19, 20, 21]. Finding them as a result of the miRNA profiling supports the high relevance of the observed miRNA variations with respect to CRC. In the other two most prominent networks, the link to cancer is more indirect for the respective genes although the score of their relevance to CRC is basically identical to that of *SMAD2/AKT* and *CDKN1A* (Table 2). The genes encoding for Neuregulin 1 (*NRG1*, Fig. 3C) and chemokine C‐X‐C ligand 12 (*CXCL12*, Fig. 3D) were identified as being strongly affected by the detected miRNA increases and decreases. Both genes are located central in their respective regulative network. NRG1 is one of four ligands (NRG 1–4) for the human epidermal growth factor receptor family (HER 1–4), which in turn is strongly implicated in the growth and spreading of cancer cells [19, 22]. CXCL12 is the main ligand of chemokine receptor 4 (CXCR4) and overexpressed in metastasis of various cancers including CRC [23, 24]. Based on the identification of genes/proteins that correspond to the mCRC‐specific miRNA patterns (Table 2), also relevant signalling pathways could be determined. This includes ‘Retinoic Acid Receptor (RAR) Activation’ and ‘Eukaryotic Initiation Factor 2 (EIF2) Signalling’; both of them are involved in transcriptional and translational gene regulation. In addition, ‘Human Embryonic Stem Cell Pluripotency’ was found as well as the directly cancer‐associated ‘Molecular Mechanisms of Cancer’, ‘Pancreatic Adenocarcinoma Signalling’ and ‘Colorectal Cancer Signalling’ (Fig. S3).

**Table 2:** List of the most strongly affected regulative networks in metastatic colorectal cancer (mCRC). A pathway analysis was performed based on the observed miRNA variations. Ranking was according to the number of miRNAs found to be involved in a network. Functional and disease annotations are given next to a score that indicates relevance; a score > 2 is considered significant. The molecules involved in the networks are listed. In bold, the regulated miRNAs are shown; the direction of change – up or down – is indicated by arrows.

Ranking	Molecules in network	Score	Focus molecules	Top diseases/functions
1	Akt, calcifediol, CFAP45, Cg, Gulo, Hif, Insulin, KRTAP20‐1, MAP2K1/2, mir‐26, mir‐126, mir‐363, OIP5‐AS1, PTCHD3, Smad2/3, Vegf, 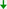 let‐7a‐5p (and miRNAs with seed GAGGUAG), 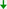 miR‐101‐3p (and ACAGUAC), 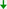 miR‐103‐3p (and GCAGCAU), 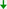 miR‐126a‐3p (and CGUACCG), 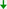 miR‐130a‐3p (and AGUGCAA), 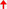 miR‐143‐3p (and GAGAUGA), 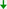 miR‐148a‐3p (and CAGUGCA), 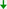 miR‐155‐5p (and UAAUCGU), 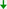 miR‐16‐5p (and AGCAGCA), 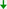 miR‐17‐5p (and AAAGUGC), 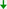 miR‐181a‐5p (and ACAUUCA), 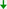 miR‐185‐5p (and GGAGAGA), 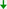 miR‐19b‐3p (and GUGCAAA), 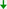 miR‐21‐5p (and AGCUUAU), 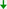 miR‐26a‐5p (and UCAAGUA), 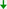 miR‐532‐5p (and AUGCCUU), 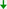 miR‐660‐5p (and ACCCAUU), 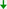 miR‐7a‐5p (and GGAAGAC), 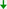 miR‐92a‐3p (and AUUGCAC)	31	19	Cancer, organismal injury and abnormalities, reproductive system disease
2	ACP1, ADAT3, BRCA2, CDKN1A, EVI2B, FMN2, HK2, IKBKB, MORN5, PIKFYVE, RHOA, RHPN2, SMC2, SSTR3, TIPRL, TMEM211, WDR73 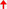 miR‐1825 (and miRNAs with seed CCAGUGC) 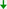 miR‐188‐3p (and UCCCCACA) 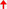 miR‐211‐3p (and CAGGGAC) 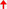 miR‐3064‐5p (and GGCUGUU) 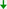 miR‐335‐5p (and CAAGAGC) 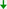 miR‐340‐5p (and UAUAAAG) 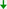 miR‐454‐5p (and CCCUAUC) 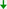 miR‐4672 (and UACACAG) 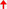 miR‐4758‐3p (and GCCCCAC) 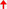 miR‐4788 (and UACGGAC) 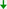 miR‐505‐5p (and GGAGCCA) 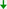 miR‐5695 (and CUCCAAG) 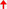 miR‐6087 (and GAGGCGG) 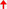 miR‐6508‐5p (and CUAGAAA) 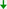 miR‐6749‐5p (and CGGGCCU) 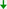 miR‐6800‐3p (and ACCUCUC) 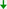 miR‐933 (and GUGCGCA)	33	17	Cancer, cell morphology, cellular assembly and organisation
3	ARMCX2, C11orf42, C11orf45, CD248,ERICH1, GALNT15, GRID2IP, GSR, KCNC2, LARGE2, MSC‐AS1, NME6, NRG1, SYT8, TEX44, TMEM258, TRANK1, ZNF608 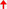 miR‐1234‐3p (and miRNAs with seed CGGCCUG) 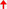 miR‐1249‐3p (and CGCCCUU) 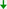 miR‐1275 (and UGGGGGA) 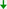 miR‐1285‐3p (and CGGCCUG) 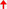 miR‐1343‐5p (and GGGGAGC) 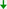 miR‐15a‐3p (and AGGCCAU) 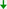 miR‐183‐5p (and AUGGCAC) 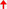 miR‐3083‐5p (and GGCUGGG) 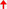 miR‐3558‐3p (and CUGUGGA) 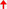 miR‐4787‐3p (and AUFCGCC) 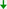 miR‐500a‐3p (and UGCACCU) 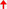 miR‐545‐5p (and CAGUAAA) 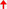 miR‐6085 (and AGGGGCU) 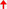 miR‐6165 (and AGCAGGA) 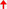 miR‐6808‐5p (and AGGCAGG) 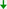 miR‐708‐5p (and AGGAGCU)	31	16	Cell cycle, DNA replication, recombination & repair, cardiovascular disease
4	AKT1, CHID1,CXCL12, DZIP1, GPR162, GPR171, GSK3B, LPAR2, MOGAT3, MORN5, MTMR7, PLCL1, PPP1R7, PRSS22, SEC14L2, SMO, STK36, THYN1 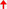 miR‐1202 (and miRNAs with seed UGCCAGC), 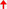 miR‐1228‐3p (and CACACCU), 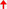 miR‐1238‐3p (and UUCCUCG), 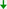 miR‐1273e (and UGCUUGA), 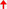 miR‐3072 (and GCCCCCU), 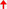 miR‐3162‐3p (and CCCUACCI), 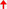 miR‐3594‐5p (and CCAGGGGC), 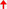 miR‐3960 (and GCGGCGG), 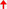 miR‐4443 (and UGGAGGC), 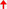 miR‐4459 (and CAGGAGG), 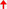 miR‐5100 (and UCAGAUC), 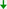 miR‐5189‐3p (and GCCAACC), 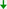 miR‐550b‐2‐5p (and UGUGCCU), 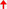 miR‐6089 (and GAGGCCG), 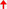 miR‐6840‐3p (and CCCAGGA), 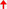 miR‐7056‐5p (and GUGGAGG)	30	16	Cancer, organismal functions, organismal injury and abnormalities
5	ATG14, CBLC, CCR10, DRD3, ERAS, GAREM1, GRB2, KRT6A, LHCGR, LINC01976, MAGIX, NPY2R, PHLDA1, POM121L12, PRR32, SALL3, SHC1, SHC4, TESK1, TFG, TOP2A 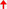 miR‐1233‐5p (and miRNAs with seed GUGGGAG), 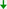 miR‐1255b‐5p (and GGAUGAG), 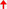 miR‐1260a (and UCCCACC), 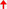 miR‐3180‐3p (and GGGGCGG), 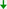 miR‐342‐5p (and GGGGUGC), 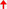 miR‐4281 (and GGUCCCG), 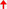 miR‐4665‐3p (and UCGGCCG), 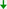 miR‐4737 (and UGCGAGG), 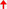 miR‐574‐5p (and GAGUGUG), 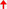 miR‐6069 (and GGCUAGG), 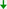 miR‐629‐5p (and GGGUUUA), 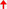 miR‐6967‐5p (and AGGGAGG), 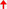 miR‐802‐3p (and CGGAGAG), 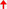 miR‐8069 (and GAUGGUU)	26	14	Cancer, organismal injury and abnormalities, reproductive system disease
6	AC1127152, CCL2, CCNG2, CSMD3, DCUN1D1, FER1L6, FN1, ICAM1, KIAA1109, ODAM, OTULIN, RPL15, RPL26, SAXO1, SENP5, SMAD2, SYT8, TP53, WNT8A, XPO5 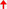 miR‐1207‐5p (and miRNAs with seed GGCAGGG), 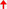 miR‐1246 (and AUGGAUU), 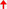 miR‐1268a (and GGGCGUG), 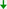 miR‐142‐5p (and AUAAAGU), 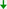 miR‐374b‐5p (and UAUAAUA), 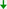 miR‐4318 (and ACUGUGG), 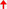 miR‐4434 (and GGAGAAG), 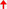 miR‐4515 (and GGACUGG), 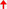 miR‐504‐5p (and GACCCUG), 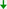 miR‐652‐3p (and AUGGCGC), 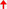 miR‐6821‐5p (and UGCGUGG), 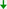 miR‐7028‐3p (and CUUCUCU), 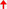 miR‐7977 (and UCCCAGC)	22	13	Cancer, gastrointestinal disease, organismal injury and abnormalities

## 4 Discussion

In this study, we compared miRNA abundance profiles in blood samples taken from patients and a cohort of healthy individuals. The study did not investigate plasma molecules but analysed the entire miRNA content of a blood sample, including that of peripheral blood mononuclear cells (PBMCs) or red blood cells. It has been suggested that the latter even act as miRNA repositories [25]. A large number of studies have documented the feasibility of this approach [e.g. 13, 14, 15]. As no analysis has yet been performed that compares plasma from blood profiles, it is difficult to evaluate to what extend these two types of miRNA profiles may be distinct or overlapping. All cells are receiving and releasing miRNAs and are therefore likely to both be affected by and contribute to the miRNA composition in plasma [e.g. 26].

To our surprise, we did not find altered miRNA levels in blood from non‐CRC patients with diverticulitis and colorectal adenomas as compared to healthy donors. The lack of any changes is remarkable, as earlier studies on various diseases – cancerous and noncancerous – did consistently identify level variations in blood [9, 13]. Why this is different for colorectal adenomas and diverticulitis requires further studies. However, the result indicates that the identified CRC‐associated miRNA alterations are not confounded by premalignant or inflammatory colorectal conditions.

Another important finding was the only small overlap of deregulated miRNAs across the three CRC stages: 152 of the 179 deregulated miRNAs (84.9%) were specific for one CRC stage only; 24 (13.4%) changed in two CRC stages. Only three (1.7% of 179 deregulated and 0.1% of all 2.549 tested miRNAs) – miR‐1225‐5p, miR‐1207‐5p and miR‐4459 – were specific for CRC at all stages. A recent meta‐analysis about 617 miRNAs, which had been reported to be deregulated in 3.454 cases of CRC, did not identify a single miRNA that was common to all CRC samples [5]. However, the studies included in the meta‐analysis relied on a mostly small number of preselected miRNAs. Increased miR‐1207‐5p levels have not yet been reported in cancer patients at all, and only one study each has mentioned increased levels of miRNA‐1225‐5p in breast cancer patients [27] and of miR‐4459 in ovarian cancer [28]. The data (Table S4) suggest that the 179 miRNAs may have functions in CRC pathobiology when circulating in blood. Particularly the stage‐specific miRNAs but also the molecules common to more than one CRC stage could have potential for blood‐based diagnostic tests as a supplement to existing CRC screening processes [2].

We considered the selection of a best performing set of miRNAs for the establishment of a miRNA signature for diagnostic purposes. While there are numerous reports, in which this was done, we actually refrained from doing so. While sensitivity and specificity values could be calculated in principle and an optimal signature be established, including even a validation in the second set of 36 samples, this would nevertheless be a more academic activity with little relevance to diagnostics. The sample size is too small for producing meaningful data in this respect. Towards a potential clinic use eventually, such an analysis is called for, of course. However, substantially more samples and a multicentre approach are required to such end.

Pathway analysis on the basis of the observed miRNA variations in the mCRC‐specific dataset identified *NRG1* and *CXCL12* as most significantly affected molecules. NRG1 binds predominantly to HER3 and HER4 [22, 31]. NRG1/HER3‐activated signalling pathways (e.g. PI3K‐AKT) promote cell proliferation, migration and survival [18, 19, 20, 21, 22, 29], while blocking of NRG1‐mediated HER3 activation inhibits migration and growth of cancer cells in preclinical models [30, 31]. NRG1‐mediated HER4 activation triggers the YAP/TAZ‐Hippo‐signalling network, which is a central regulator of cells size, structure, shape and polarity [31, 32, 33, 34]. NRG1 is also known to activate tumour‐associated epithelial cells, thereby facilitating metastasis [19]. CXCL12 induces cell migration in inflammation and promotes epithelial–mesenchymal transition (EMT) as well as stem‐like features that provide phenotype plasticity [23, 24]. Other identified molecules were RAR and EIF2, which function as gene expression regulators [34, 35], SMAD2 and AKT, which play several roles in CRC tumour biology including promotion of EMT [16, 17, 18], and the cell cycle regulator p21, which is encoded by the gene *CDKN1A* and involved in the regulation of cell division, differentiation and migration [19, 20, 21]. Since IPA provides cause–effect relationships rather than mere associations [12], targets being identified here may promote haematogenous CRC dissemination, making them to candidates for predicting, preventing or treating mCRC disease.

The blood circulation is crucial for CRC dissemination, which is powered by circulating tumour cells (CTCs) as potential seeds [36]. However, only a small CTC fraction of about 0.01% with a distinct phenotype can survive in the blood circulation and give rise to distant metastasis [37]. miRNAs contribute to relevant CTC programming, which could increase plasticity, motility, mobility, invasiveness and migratory traits required for intra‐ and extravasation [38]. miRNAs are emerging as mediators of intercellular communication to reprogram recipient cells and seem to play a key role for premetastatic niche formation at distant organ sites [39, 40]. It is an interesting hypothesis that the mCRC‐specific miRNAs in blood may function as paracrine mediators in cell‐to‐cell signalling targeting the CTC population. Uptake of miRNAs could expand the CTC fraction capable of haematogenous spread. In support of this hypothesis, all affected proteins predicted on the basis of our mCRC‐specific deregulated miRNA dataset, most significantly *NRG1* and *CXCL12*, are part of the pathways involved in cancer signalling and stem cell pluripotency.

## 5 Conclusions

This study provides leads for improved biomarker discovery for CRC screening, but it also widens the horizon for the design of novel antimetastatic therapies. Since our results have been obtained by an exploratory single‐centre analysis, they warrant confirmatory testing in an independent multicentre validation. Further, clinical and functional studies will be required to determine precisely the origin and function of miRNA alterations in blood and to verify predicted causality relations. However, this is the first study providing blood expression profiles in CRC that is representative of the whole known human miRNA landscape. Moreover, it is first in providing a comprehensive assessment of miRNA alterations in blood at different CRC stages. The altered miRNA profiles were controlled for non‐CRC colorectal confounders and selected by comparison to healthy controls defined by inconspicuous colonoscopy.

## Conflict of interest

The authors declare no conflict of interest.

## Author contributions

AS, JH and AB designed the study, edited the data and wrote the manuscript. HW, ML and KJO organised clinical samples and contributed to data interpretation and manuscript editing. AB and CZ performed RNA profiling and the subsequent bioinformatics analyses. All authors contributed to data analysis and interpretation and approved the final version of the manuscript for publication.

### Peer Review

The peer review history for this article is available at https://publons.com/publon/10.1002/1878‐0261.13065.

## Supporting information

**Fig. S1**. IPA maps of the top four signalling pathways that correspond to the observed miRNA variations in stage I/II CRC.

**Fig. S2**. IPA map of the signalling pathway that corresponds to the observed miRNA variations in stage III CRC.

**Fig. S3**. Map of the signalling pathways that correspond to the observed miRNA variations in stage IV CRC (mCRC).

**Table S1**. Blood‐derived miRNAs that were down or up in abundance in colorectal cancer patients of UICC stage I/II (n = 16) compared with healthy controls (n = 16).

**Table S2**. Blood‐derived miRNAs that were down or up in abundance in colorectal cancer patients of UICC stage III (n = 16) compared with healthy controls (n = 16).

**Table S3**. Blood‐derived miRNAs that were down or up in abundance in colorectal cancer patients of UICC stage IV (n = 16) compared with healthy controls (n = 16).

**Table S4**. Protein molecules that are probably affected by the miRNAs exhibiting varying blood levels.

## Data Availability

The data are available in the Gene Expression Omnibus (GEO) data repository under accession number GSE18020. The data will be also online at https://www.dkfz.de/funct_genome/data‐of‐submitted‐manuscript.html.
