# Supplementary figures and images for: MicroRNAs in blood act as biomarkers of colorectal cancer and indicate potential therapeutic targets
Source: Mol Oncol. 2021 Aug 5;15(9):2480–90. doi: 10.1002/1878-0261.13065 (PMC8410571; doi:10.1002/1878-0261.13065)

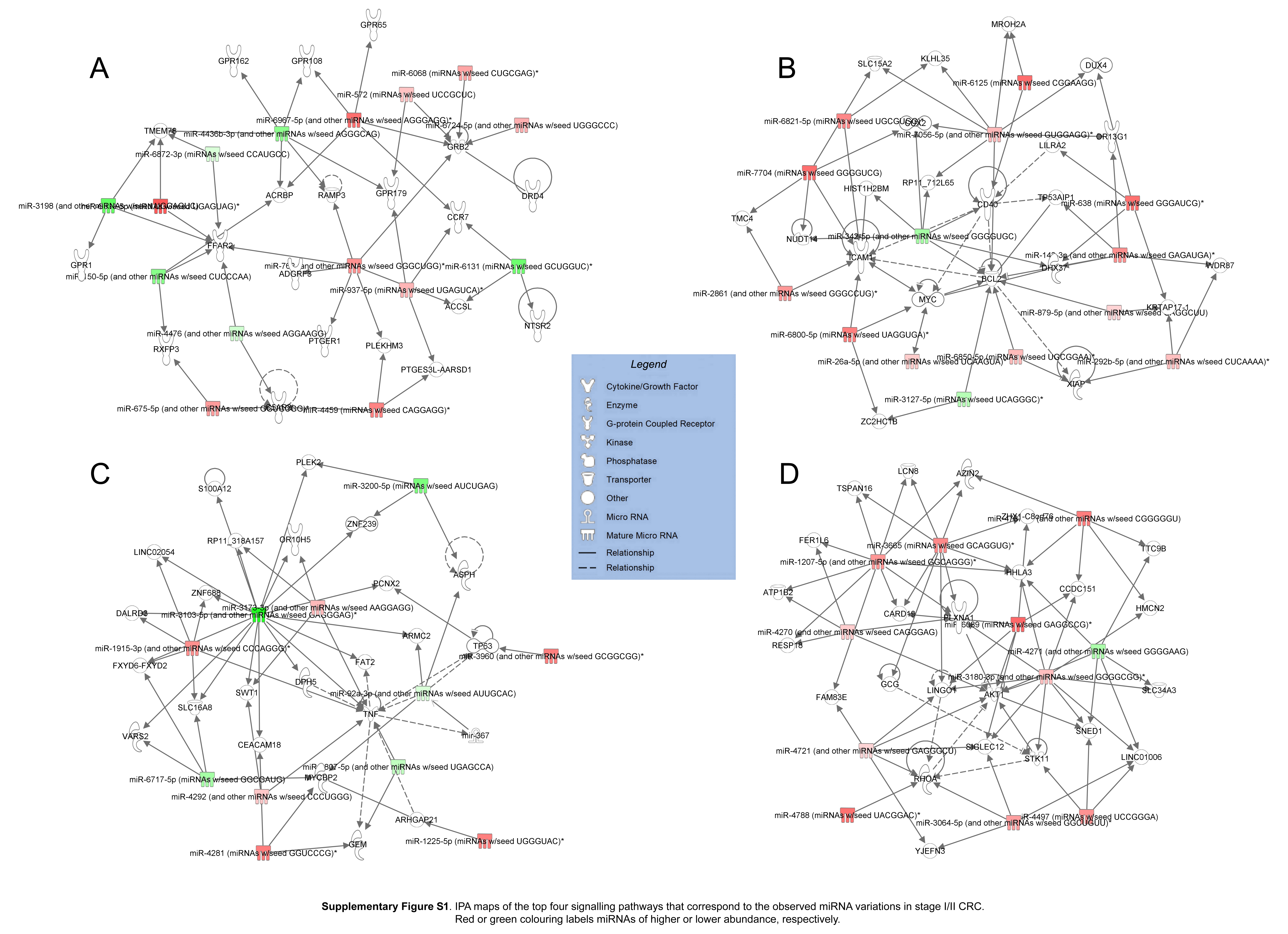

Supplement: Supplementary file 1 — Fig. S1. IPA maps of the top four signalling pathways that correspond to the observed miRNA variations in stage I/II CRC. [file MOL2-15-2480-s005.jpg]

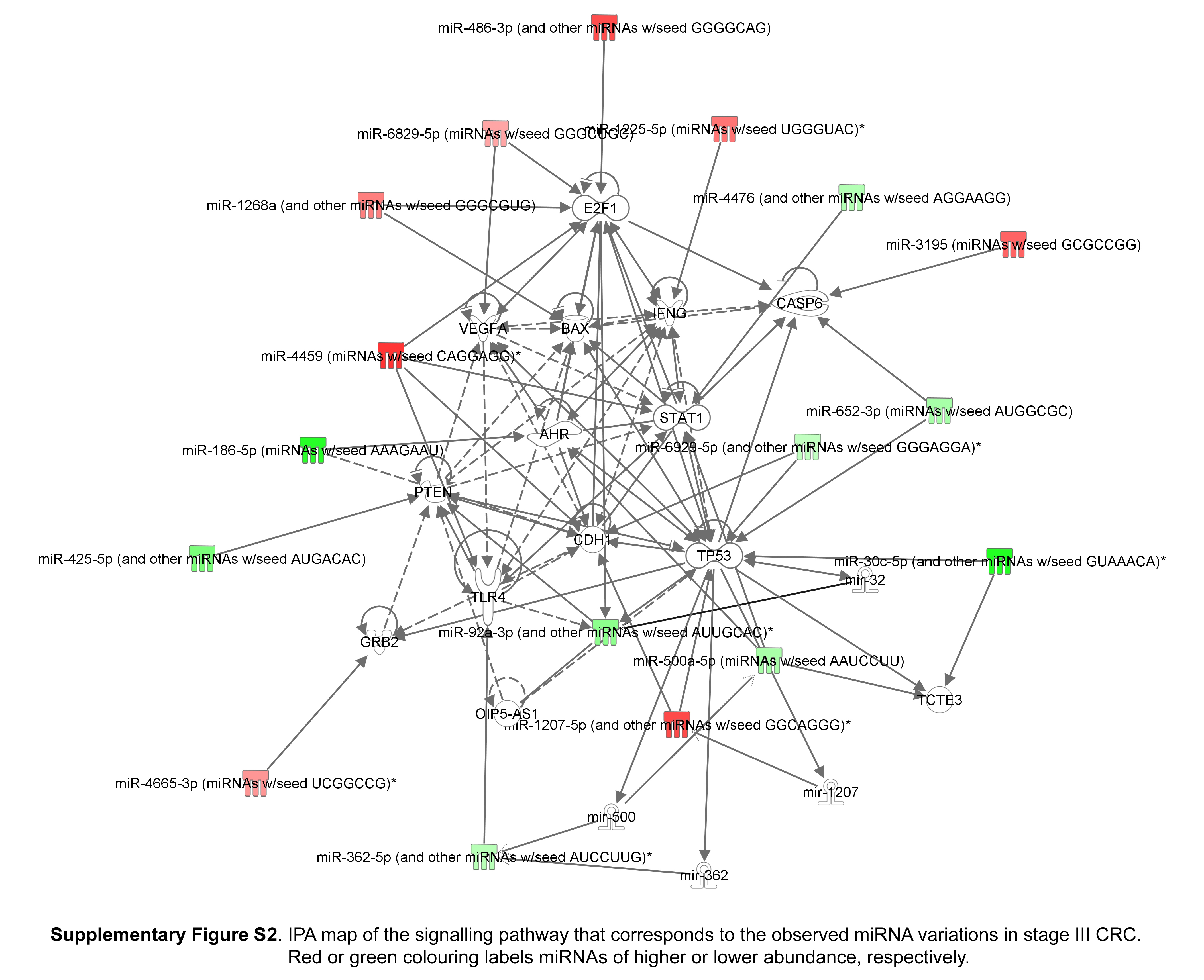

Supplement: Supplementary file 2 — Fig. S2. IPA map of the signalling pathway that corresponds to the observed miRNA variations in stage III CRC. [file MOL2-15-2480-s001.jpg]

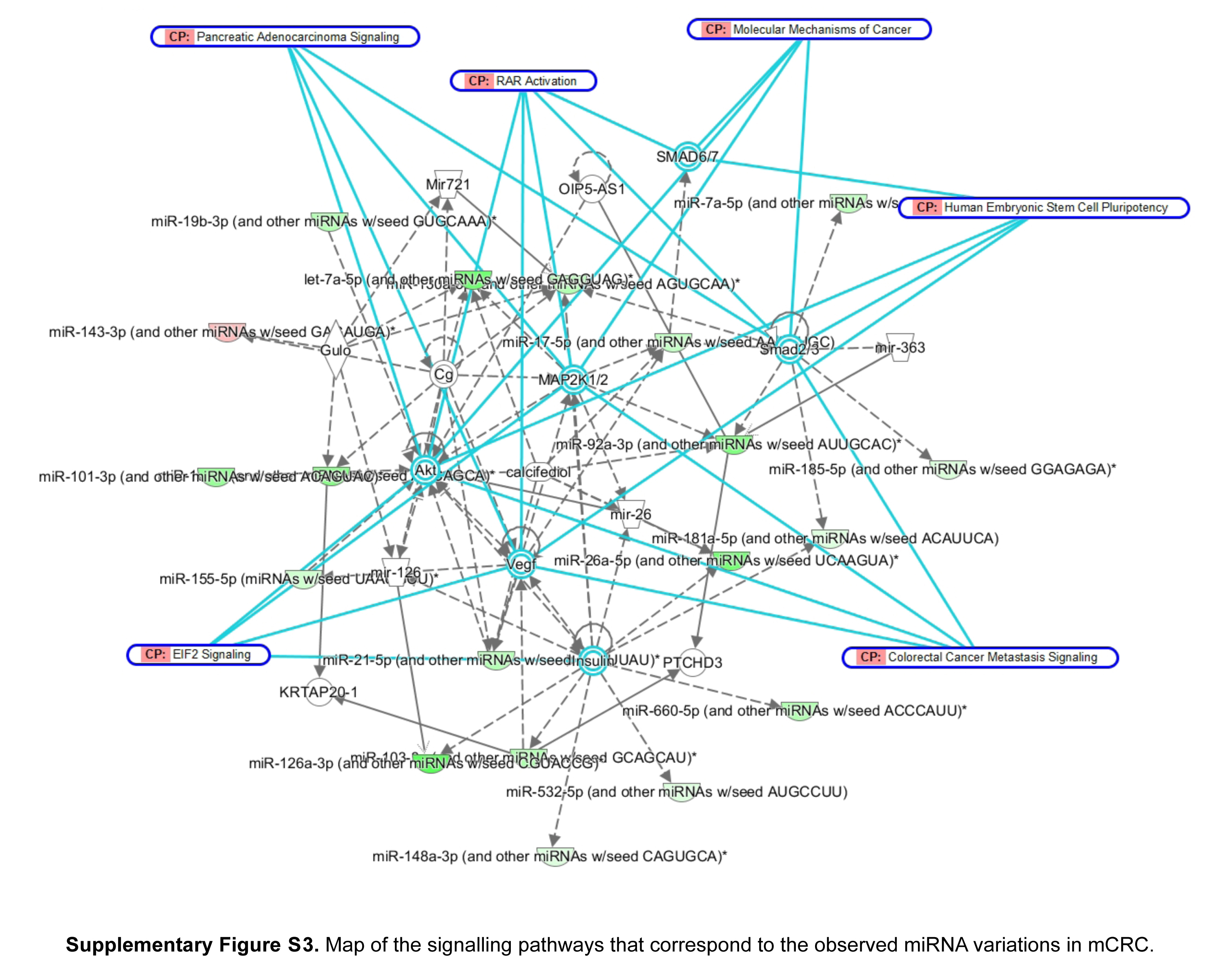

Supplement: Supplementary file 3 — Fig. S3. Map of the signalling pathways that correspond to the observed miRNA variations in stage IV CRC (mCRC). [file MOL2-15-2480-s003.jpg]
